# *Iris Pallida* Extract Alleviates Cortisol-Induced Decrease in Type 1 Collagen and Hyaluronic Acid Syntheses in Human Skin Cells

**DOI:** 10.3390/cimb45010025

**Published:** 2023-01-01

**Authors:** Jung Ha Choo, Hong Gu Lee, So Young Lee, Nae Gyu Kang

**Affiliations:** Science Research Park, LG Household and Healthcare Ltd., Gangseo-gu, Seoul 07795, Republic of Korea

**Keywords:** *Iris pallida*, glucocorticoids, cortisol, glucocorticoid receptor, collagen, hyaluronic acid, stress

## Abstract

Excessive endogenous or exogenous levels of the stress hormone cortisol have negative effects on various tissues, including the skin. *Iris pallida* (IP), used in traditional medicine and perfumes, exhibits biological activities, such as antioxidant and anti-inflammatory activities. In this study, we aimed to investigate the inhibitory effect of IP extract (IPE) on cortisol activity in human skin cells. We found that IPE alleviated the cortisol-induced decrease in the levels of procollagen type 1 and hyaluronic acid (HA), which were significantly recovered by 106% and 31%, respectively, compared with cortisol-induced reductions. IPE also rescued the suppression of the gene expression of *COL1A1* and the HA synthases *HAS2* and *HAS3* in cortisol-exposed cells. Moreover, IPE blocked the cortisol-induced translocation of the glucocorticoid receptor (GR) from the cytoplasm to the nucleus as effectively as the GR inhibitor mifepristone. Analysis using a high-performance liquid chromatography–diode-array detector system revealed that irigenin, an isoflavone, is the main component of IPE, which restored the cortisol-induced reduction in collagen type 1 levels by 82% relative to the cortisol-induced decrease. Our results suggest that IPE can act as an inhibitor of cortisol in human skin cells, preventing cortisol-induced collagen and HA degradation by blocking the nuclear translocation of the GR. Therefore, IPE may be used as a cosmetic material or herbal medicine to treat stress-related skin changes.

## 1. Introduction

Cortisol is a glucocorticoid (GC) hormone that is mostly synthesized and secreted in the cortex of the adrenal gland in response to diverse stimuli, including psychological and physiological stress. GC production and release are tightly controlled by the hypothalamic–pituitary–adrenal (HPA) axis. In response to stress stimuli, the hypothalamus secretes corticotrophin-releasing hormone (CRH), which stimulates the pituitary gland to produce and release adrenocorticotropic hormone (ACTH). Subsequently, ACTH triggers the synthesis and release of GCs from the cortex of the adrenal gland [1]. GCs are involved in various physiological functions such as growth, development, reproduction, metabolism, water balance, cardiovascular function, immune responses, and cognitive functions [2]. Most GC actions are mediated via the glucocorticoid receptor (GR), a member of the nuclear receptor superfamily encoded by the nuclear receptor subfamily 3, group C, member 1 (*NR3C1*) gene [3]. When GCs bind to the GRs, the GC-GR complexes are translocated from the cytoplasm to the nucleus and directly interact with glucocorticoid-responsive elements (GREs) located in the promoter regions of target genes and/or physically linked with other transcription factors, thereby regulating the transcription of target genes [4].

The skin, the largest organ in the human body, comprises multiple layers: the epidermis, dermis, and subcutaneous tissues. The extracellular matrix (ECM) occupies the space between cells and accounts for the largest portion of the skin [5]. Collagen is the main ECM protein found in various connective tissues of the body, maintaining structural integrity and playing various physiological roles [6]. Collagen type 1 is the most abundant collagen in the body and is composed of heterotrimers with 2 α1 chains and 1 α2 chain, encoded by *COL1A1* and *COL1A2*, respectively [7,8]. Hyaluronic acid (HA), also known as hyaluronate or hyaluronan, is another major constituent of the ECM of the skin. HA is a non-sulfated glycosaminoglycan comprising repeating disaccharides of d-glucuronic acid and *N*-acetyl-d-glucosamine [9]. HA is present not only in the dermis, but also the epidermal intercellular spaces, especially the middle spinous layer [10,11,12,13]. Moreover, HA is found in the basal layer of the spinous layer, as well as in the stratum corneum [14]. HA is synthesized by HA synthase (HAS), mainly in the fibroblasts and keratinocytes of the skin. Three isoforms of HAS (HAS1, HAS2, and HAS3) have been identified in humans [15].

The skin acts as a defensive barrier to protect the inner tissues against external environmental stressors, such as mechanical and physical damage, UV light, hazardous chemicals, and microorganisms. A peripheral HPA axis equivalent to the central HPA axis exists in the skin [16,17], mediating cortisol production [18,19,20]. Under chronic psychological stress, high levels of GCs are continuously released, which have detrimental effects on the skin, such as the increased risk of infections owing to immunosuppression, tissue atrophy, and impaired wound healing [21,22,23,24]. Excessive endogenous cortisol synthesis or exogenous GC application leads to skin atrophy, which involves flat dermal–epidermal junctions, skin thinning, reduced number of fibroblasts, small keratinocytes, and reduced collagen and HA production [23,24,25,26]. Thus, the inhibition of GCs may prevent or reverse psychological-stress-induced skin changes [26].

*Iris pallida* is a perennial herbaceous plant belonging to the family Iridaceae. It is native to Dalmatia in Croatia; however, its distribution extends to Europe, the Middle East, Africa, and North America [27]. The dried rhizome of *I. pallida*, along with that of *I. germanica* and *I. florentina*, is known as *rhizome iridis* or orris root. This orris root has been used as a source of essential oil and is widely used in the cosmetic, perfume, and food industries [28]. To obtain high-quality essential oil from orris root, the rhizomes must be aged for at least 3 years because the violet-like odor compounds, irones, are fully developed by gradual oxidative degradation [29]. Several studies have been conducted on the chemical composition of essential oils and extracts of *I. pallida* rhizomes [30,31,32,33]. The constituents of the essential oil of *I. pallida* are fatty acids, aromatic compounds, alkanes, sesquiterpenes, and triterpenes [29], and the extracts of *I. pallida* contain phenolic compounds such as isoflavone, isoflavanone, and benzophenone [30,31,32]. Although various molecules isolated from *Iris* spp. exhibit many biological activities, such as anti-inflammatory, antibacterial, antioxidant, antimutagenic, and anticancer activities [33,34,35,36,37], there are few studies on the biological activities of the extract or compounds derived from *I. pallida* rhizome, particularly their activity in human skin cells. In the present study, we aimed to investigate the inhibitory effect of *I. pallida* extract (IPE) on the cortisol-induced decrease in collagen and HA levels in human skin cells.

## 2. Methods

### 2.1. Preparation of Iris pallida Extract

The *Iris pallida* extract (IPE) used in this study was a solution in which ORRIS RESOID LMR (151432) supplied by LMR Naturals by Iff (Grasse, France) was diluted in dipropylene glycol (DPG). LMR Naturals by Iff certify that this ORRIS RESOID LMR was extracted from *I. pallida* roots containing rhizomes cultivated in Italy and aged for at least 3 years. According to the manufacturer’s process diagram, *I. pallida* roots were harvested, dried, ground, extracted with a solvent, and purified with ethyl alcohol.

### 2.2. Cell Culture

Human immortalized keratinocytes (HaCaTs) were obtained from AddexBio (San Diego, CA, USA) and human dermal fibroblasts (HS68s) were purchased from the American Type Culture Collection (ATCC; Manassas, VA, USA). All cells were cultured using Dulbecco’s modified Eagle’s medium (DMEM; Gibco, Thermo Fisher Scientific, Waltham, MA, USA) supplemented with 1% antibiotic–antimycotic solution (Gibco) and 10% fetal bovine serum (FBS; Gibco) in a humidified atmosphere with 5% CO_2_ at 37 °C.

### 2.3. Cell Viability Assay

Cell viability was assessed using a cell counting kit-8 assay (CCK-8; Dojindo Molecular Technologies, Rockville, MD, USA). Cells (1 × 10^4^) were plated in 96-well plates and cultured for 24 h. The next day, the cells were washed with phosphate-buffered saline (PBS; Solbio, Seoul, Republic of Korea) and treated with various concentrations of IPE or irigenin in serum-free DMEM with or without cortisol (Sigma-Aldrich, St. Louis, MO, USA) for 48 h. Next, a 10% CCK-8 solution was added to the medium, and the cells were incubated for 2 h. The absorbance of the samples was measured at 450 nm using a SYNERGY H1 microplate reader (BioTek, Winooski, VT, USA).

### 2.4. Enzyme-Linked Immunosorbent Assay 

HS68 or HaCaT cells (1.5 × 10^5^ cells/well) were seeded in 24-well plates and cultured for 24 h. The cells were washed with PBS and treated with serum-free DMEM containing either vehicle or IPE for 1 h, followed by 1 μM cortisol induction. After 48 h of incubation, the cell culture medium was collected and the levels of procollagen type 1 αl and HA were determined using the Human Procollagen I Alpha 1 and Hyaluronan DuoSet ELISA kits (R&D Systems, Minneapolis, MN, USA), respectively, according to the manufacturer’s protocol. Irigenin treatment of HS68 cells and ELISA for procollagen type 1 αl were performed in the same manner as described above.

### 2.5. RNA Extraction and Real-Time Quantitative Reverse-Transcription Polymerase Chain Reaction 

HaCaT (1.5 × 10^5^ cells/well) and HS68 cells (3 × 10^5^ cells/well) were plated in 24- and 12-well culture plates, respectively, and incubated for 24 h. The cells were washed with PBS, treated with serum-free DMEM containing either vehicle or IPE for 1 h, and stimulated with cortisol for 24 h. The total RNA was extracted from HS68 and HaCaT cells using the RNeasy mini kit (QIAGEN GmbH, Hilden, Germany) according to the manufacturer’s protocol. The RNA concentration and purity were measured using a NanoDrop 2000 spectrophotometer (Thermo Fisher Scientific, Waltham, MA, USA). Five hundred nanograms of RNA were reverse-transcribed using a cDNA synthesis kit (Philekorea, Seoul, Republic of Korea), and qRT-PCR was performed using the StepOnePlus^®^ Real-Time PCR System (Applied Biosystems, Waltham, MA, USA). The following TaqMan probes were used for qRT-PCR: *Col1A1* (Hs00164004_m1), *HAS2* (Hs00193435_m1), *HAS3* (Hs00193436_m1), and glyceraldehyde-3-phosphate dehydrogenase (*GAPDH*; #4333764F; Thermo Fisher Scientific, Waltham, MA, USA). The data were analyzed using the 2^–∆∆Ct^ method, and the relative expression of each gene was normalized to *GAPDH* expression.

### 2.6. Immunofluorescence Analysis

HaCaT cells (2 × 10^4^ cells/well) were plated in a 24-well plate and cultured for 24 h. The cells were rinsed with PBS; treated with a medium containing vehicle, IPE, or GR inhibitor (mifepristone; Sigma-Aldrich, St. Louis, MO, USA) for 1 h; and stimulated with cortisol for 1 h. The cells were fixed with 4% formaldehyde solution (Biosesang, Seongnam, Republic of Korea), permeabilized with 0.1% Triton X-100, and blocked with 5% FBS and 1% BSA solution for 1 h. The cells were then sequentially incubated with polyclonal anti-rabbit antibody against the GR (1:20; Abcam, Cambridge, U.K.) and Alexa Fluor™ 568 goat anti-rabbit antibody (1:1000; Thermo Fisher Scientific, Waltham, MA, USA). The nuclei of HaCaT cells were stained with 1 μg/mL 4′,6-diamidino-2-phenylindole (DAPI; Sigma-Aldrich, St. Louis, MO, USA), and immunofluorescence was examined using the EVOS TM FL Auto2 imaging system (Thermo Fisher Scientific, Waltham, MA, USA). GR translocation was analyzed using images merged with GR and DAPI.

### 2.7. High-Performance Liquid Chromatography 

IPE was quantitatively analyzed using an HPLC-diode array detection (DAD) system (Agilent 1260 Infinity LC System; Agilent Technologies, Inc., Santa Clara, CA, USA). Irigenin, purchased from PhytoLab GmbH & Co., KG (Greuth, Germany), was used as the standard. IPE and irigenin were diluted in 99.9% methyl alcohol, sonicated for 30 min in an ultrasonic bath at 25–30 °C (room temperature), and filtered through a 0.45 μm pore-size syringe filter. One microliter of each sample was injected into an HPLC column (Poroshell 120 EC-C18 column, 50 mm × 4.6 mm, 2.7 μm; Agilent Technologies) at a flow rate of 1 mL/min at 40 °C. Elution was performed with 0.1% phosphoric acid in water (A) and acetonitrile (B) with gradient elution of 10% B for 0–2 min and 10%–70% B for 2–20 min, and the eluate was monitored at 269 nm.

### 2.8. Statistical Analysis

All data are presented as the mean ± the standard deviation of three independent experiments. Statistical comparisons between groups were performed using Student’s *t*-test in Microsoft Excel. Statistical significance was set as *p* < 0.05.

## 3. Results

### 3.1. IPE Is Safe for Human Dermal Fibroblasts and HaCaT Cells

**Figure 1:**
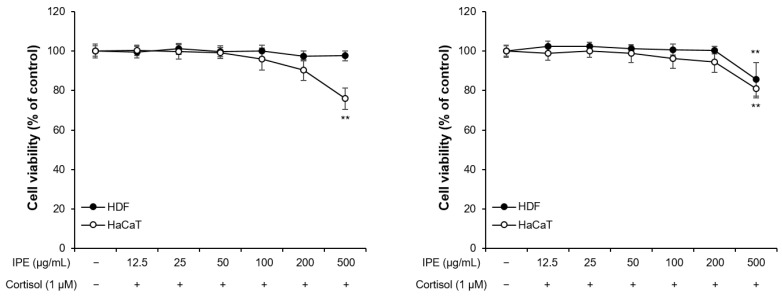
Effects of *Iris pallida* extract (IPE) on the viability of HDFs and HaCaT cells. The cells were treated with IPE at various concentrations (12.5, 25, 50, 100, 200, and 500 μg/mL) for 48 h with or without cortisol (1 μM). Cell viability was assessed using the cell counting kit (CCK)-8 assay. ** *p* < 0.01 compared to the control.

To determine whether IPE is cytotoxic to human skin cells, we treated human dermal fibroblasts (HDFs) and HaCaT keratinocytes with varying concentrations of IPE (12.5, 25, 50, 100, 200, and 500 μg/mL) with or without cortisol (1 μM) and assessed cell viability using the CCK-8 assay. Our results showed that IPE was not toxic to HDFs or HaCaT cells up to a concentration of 200 μg/mL in the presence or absence of cortisol (Figure 1). Therefore, IPE is safe for human skin cells.

### 3.2. IPE Alleviates Cortisol-Induced Reduction in Collagen Type 1 in HDFs

**Figure 2:**
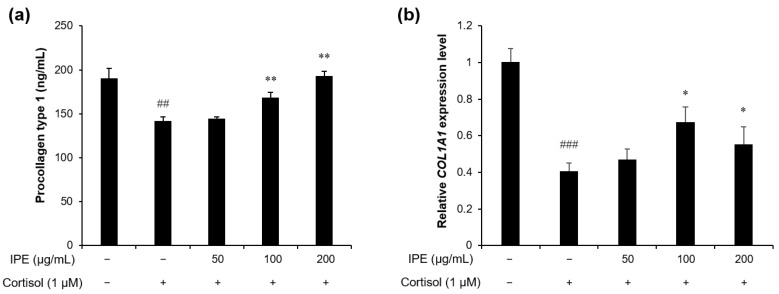
Effects of IPE on collagen type 1 synthesis in cortisol-exposed HDFs. The cells were pretreated with IPE (50, 100, and 200 μg/mL) and, then, treated with cortisol (1 μM) for 48 h. (**a**) Enzyme-linked immunosorbent assay of procollagen type 1 level in the culture supernatant of HDFs. (**b**) Real-time quantitative reverse-transcription polymerase chain reaction of relative *COL1A1* expression levels. ## *p* < 0.01, ### *p* < 0.001 compared to the control that was not exposed to cortisol; * *p* < 0.05, ** *p* < 0.01, compared to the cortisol-exposed control.

To investigate the effect of IPE on the cortisol-induced reduction in collagen synthesis, HDFs were pretreated with various concentrations of IPE (50, 100, and 200 μg/mL) and, then, exposed to cortisol. We measured procollagen type I production in the culture medium using ELISA. Our results revealed that the procollagen type 1 level in the cortisol-treated group was significantly decreased compared with that in the control group, which was treated with the vehicle. However, IPE rescued the cortisol-induced inhibition of procollagen type 1 synthesis in a dose-dependent manner (Figure 2a). At a concentration of 200 µg/mL, IPE significantly recovered the procollagen type 1 level by 106% compared with cortisol-induced reduction, which was similar to the level of the control group treated with vehicle only.

To confirm the effect of IPE on collagen production, we analyzed the transcription level of *COL1A1* in HDFs by performing q-RT-PCR. The results revealed that the reduced *COL1A1* mRNA expression in cortisol-stimulated HDFs was rescued by treatment with IPE (Figure 2b). Collectively, our findings showed that IPE prevents cortisol-induced downregulation of collagen synthesis.

### 3.3. IPE Inhibits Cortisol-Induced Reduction in HA Production in HaCaT Cells

**Figure 3:**
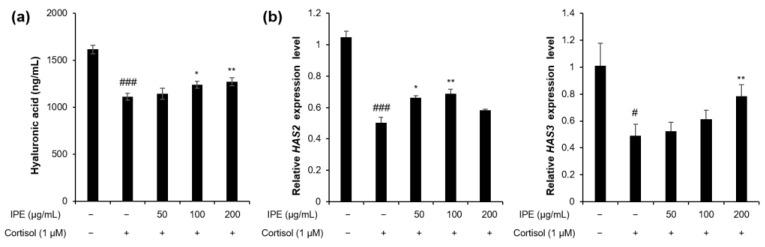
Effects of IPE on hyaluronic acid (HA) synthesis in cortisol-exposed HaCaT cells. Cells were pretreated with IPE (50, 100, and 200 μg/mL) for 1 h and, then, exposed to cortisol (1 μM) for 48 h. (**a**) Enzyme-linked immunosorbent assay of HA level in the culture supernatant of HaCaT cells. (**b**) Real-time quantitative reverse-transcription polymerase chain reaction for determining the relative expression of *HAS2* and *HAS3*. # *p* < 0.05, ### *p* < 0.001 compared to the control that was not exposed to cortisol; * *p* < 0.05, ** *p* < 0.01, compared to the cortisol-exposed control.

To investigate the effect of IPE on the cortisol-induced decrease in HA production, we measured the level of HA in the culture medium of HaCaT cells that were pretreated with IPE at various concentrations (50, 100, and 200 μg/mL) followed by exposure to cortisol. The HA level was considerably reduced in the cortisol-exposed culture medium compared to the culture medium not treated with cortisol. However, the cortisol-induced decrease in HA production significantly recovered upon treatment with IPE-treated culture supernatant at 100 (*p* < 0.05) and 200 μg/mL (*p* < 0.01), by 25% and 31%, respectively (Figure 3a). We also investigated the expression levels of the enzyme-coding genes *HAS2* and *HAS3* in cortisol-exposed HaCaT cells. Compared with that in control HaCaT cells untreated with cortisol, the expression of *HAS2* and *HAS3* decreased in cortisol-treated HaCaT cells. In contrast, IPE alleviated the cortisol-induced reduction in *HAS2* and *HAS3* mRNA expression (Figure 3b).

### 3.4. IPE Inhibits Cortisol-Induced GR Nuclear Translocation

**Figure 4:**
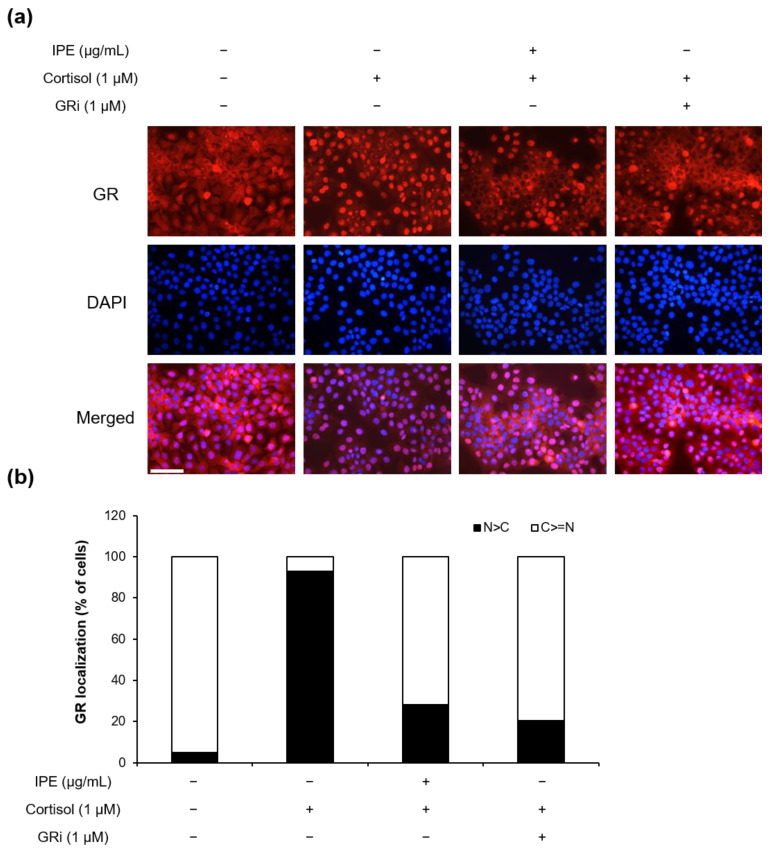
Inhibitory effect of IPE on cortisol-induced glucocorticoid receptor (GR) nuclear translocation. (**a**) Immunofluorescence analysis for GR localization in HaCaT cells. Cells were incubated in serum-free DMEM with IPE (100 μg/mL) or GR inhibitor (mifepristone, 1 μM) for 1 h before exposure to cortisol (1 μM). After 1 h of stimulation, the cells were fixed in 4% formaldehyde, permeabilized with 0.1% Triton X-100, and stained with antibody against the GR (red). DAPI (blue) was used for nuclear staining. Scale bar = 100 nm. (**b**) Quantification of GR subcellular distribution. Predominantly nuclear or cytoplasmic GR localization was analyzed using merged images with DAPI, and the number of cells according to GR location is expressed as a percentage. N, nucleus; C, cytoplasm; GRi, GR inhibitor.

To investigate whether IPE acts as an inhibitor of the GR, we performed immunofluorescence analysis of the translocation of the GR from the cytosol to the nuclei of HaCaT cells treated with 100 μg/mL IPE or 1 µM GR inhibitor (mifepristone) under cortisol stimulation. As shown in Figure 4, exposure to cortisol highly promoted GR nuclear translocation compared with that in the untreated control; however, IPE inhibited the cortisol-induced nuclear translocation of the GR to a level similar to that observed with mifepristone. This result indicates that IPE inhibits the cortisol-induced translocation of the GR from the cytoplasm to the nuclei in HaCaT cells.

### 3.5. Irigenin, the Main Component of IPE, Restores Cortisol-Induced Reduction in Collagen Type 1 Level in HDFs

**Figure 5:**
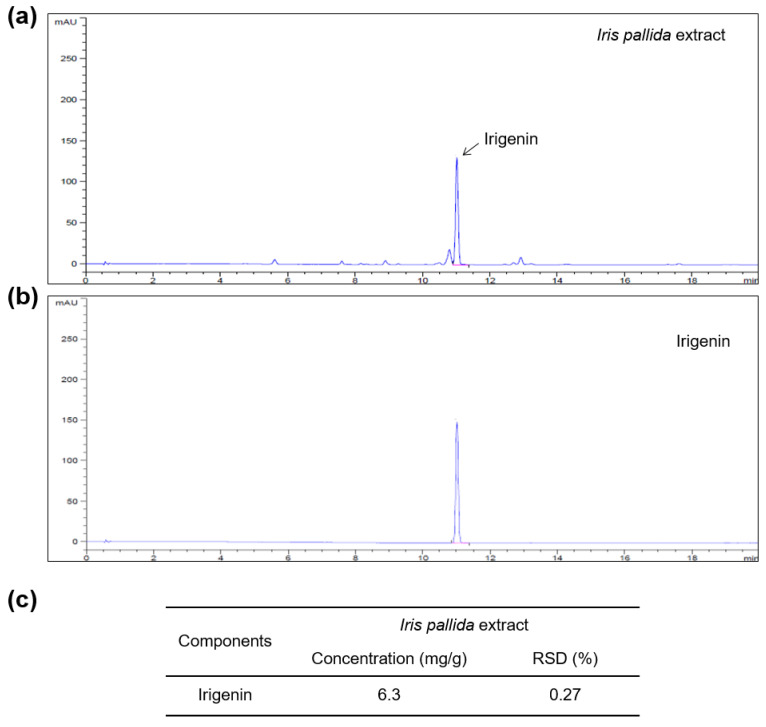
Phytochemical analysis of IPE. (**a**,**b**) High-performance liquid chromatography-diode array detection chromatograms of IPE with irigenin as the standard. (**c**) Quantification of irigenin identified in IPE. RSD, relative standard deviation.

To determine the IPE components that are involved in cortisol inhibition, we conducted a phytochemical analysis of IPE. According to a previous study, *I. pallida* resinoids are rich in flavonoids, of which irigenin is the most abundant [32]. Therefore, to identify and quantify irigenin in IPE, we performed an HPLC-DAD analysis. As shown in Figure 5, we confirmed that irigenin is the main component of IPE, accounting for 0.63% of the total content of IPE.

**Figure 6:**
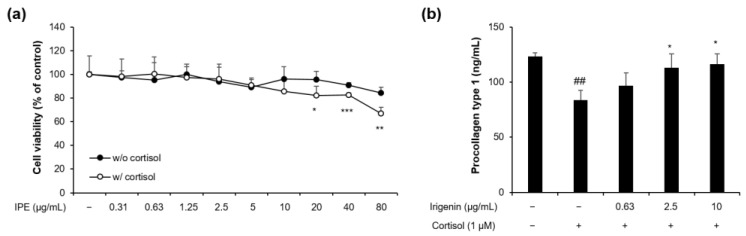
Effects of irigenin on the viability of HDFs with or without cortisol and on procollagen type 1 in cortisol-exposed HDFs. (**a**) The cells were treated with irigenin at various concentrations from 0.0315 to 80 μg/mL for 48 h with or without cortisol (1 µM). Cell viability was assessed using the CCK-8 assay. (**b**) The cells were pretreated with irigenin (0.63, 2.5, and 10 μg/mL) for 1 h and, then, exposed to cortisol (1 µM) for 48 h. ELISA of procollagen type 1 in the culture supernatant of HDFs. ## *p* < 0.01 compared to the control not exposed to cortisol; * *p* < 0.05, ** *p* < 0.01, *** *p* < 0.001 compared to the cortisol-exposed control.

As irigenin was the most-abundant constituent of IPE, we investigated whether it mediates the inhibitory effect of cortisol by evaluating the changes in procollagen type 1 level in HDFs treated with cortisol and irigenin. The treatment concentration of irigenin was determined based on the ratio of irigenin in the IPE and cell viability of irigenin treatment in HDFs (Figure 6a). Irigenin restored the cortisol-induced reduction in procollagen type 1 synthesis by 74% and 82% at 2.5 µg/mL and 10 µg/mL, respectively, in a dose-dependent manner (Figure 6b), suggesting that irigenin is the active molecule of IPE.

## 4. Discussion

Cortisol is a classic stress hormone in humans; it is synthesized and released from the adrenal cortex through the activation of the HPA axis in response to psychological stress. Chronic psychological stress induces the upregulation of cortisol in the blood, causing various physical alterations in the peripheral tissues, including the skin. In particular, the cutaneous manifestations observed in GC-induced skin atrophy are similar to the characteristics of skin aging [22,38]. Therefore, the cell culture system under high levels of cortisol can be used to mimic psychological stress conditions in vitro. Although several studies have used dexamethasone, a synthetic glucocorticoid, to mimic mental stress in vitro [39,40], in this study, we selected cortisol instead of dexamethasone to mimic the environment of the human body. Using this in vitro system, we investigated whether IPE is effective in inhibiting cortisol activation in skin cells and preventing psychological-stress-induced skin aging. We revealed the inhibitory effects of IPE on cortisol-induced skin damage by evaluating the collagen and HA levels and the expression of genes related to collagen and HA syntheses in cortisol-treated human skin cells. The immunofluorescence analysis showed that IPE attenuated the nuclear translocation of the GR. We also found that irigenin is the main component of IPE, which inhibits cortisol-induced collagen degradation.

The addition of cortisol to human fibroblasts reduces the levels of procollagen type 1 protein and mRNA by regulating the promoter activity of *COL1A1* [24,41]. This degradation of collagen in fibroblasts weakens the structural support of the skin, resulting in loss of volume and firmness, thinning, and wrinkling [42]. In the present study, we found that IPE rescued the cortisol-induced decrease in procollagen type 1 level and *COL1A1* expression levels in HDFs (Figure 2). Moreover, irigenin, the major constituent of IPE, alleviated the cortisol-induced reduction in collagen (Figure 6b). We determined the concentration of irigenin treatment based on the ratio of irigenin content in the IPE and the cell viability of irigenin in HDFs. IPE has a clear inhibitory effect on collagen level reduction by cortisol at a concentration of 100 µg/mL; the irigenin content in 100 µg/mL of IPE was established as 0.63 µg/mL based on our HPLC analysis (Figure 5). We therefore selected the starting concentration of 0.63 µg/mL irigenin for cortisol inhibition. The maximum concentration of irigenin was determined to be 10 µg/mL because cell cytotoxicity was observed at a concentration of 20 µg/mL or more of irigenin (Figure 6a). The data show that both IPE and irigenin significantly recovered procollagen type 1 levels in a dose-dependent manner. In particular, 200 µg/mL of IPE rescued procollagen type 1 levels to a degree similar to that of the control group treated with vehicle only. Irigenin also had an inhibitory effect on cortisol-dependent reduction of procollagen type 1 at all treatment concentrations. Therefore, these results suggest that IPE and irigenin may prevent skin aging by rescuing the reduction in collagen type 1 mRNA and protein levels in the skin under prolonged psychological stress conditions.

Recently, it has been reported that β-ionone, an aromatic compound, attenuates dexamethasone-induced suppression of collagen [39]. Dexamethasone is a synthetic glucocorticoid, and its action is mediated by the GR. Although we could not directly compare these results to ours, it seems that β-ionone has an effect similar to our IPE or irigenin on the inhibition of procollagen type 1 secretion mediated by GCs in HDFs.

Proper skin hydration is essential for maintaining healthy skin. HA is the key molecule involved in skin moisture because of its unique capacity to bind up to 1000-times its weight in water [43]. Excessive GC levels result in a decrease in the level of HA in the dermis and dermal fibroblasts by suppressing *HAS2* mRNA expression [44,45]. Exogenous GC also reduces HA levels in the cell culture medium, as well as *HAS2* expression levels in both epidermis and keratinocytes [46]. Moreover, the cortisol-induced decrease in HA level and *HAS* expression can result in dehydration of the human skin. In our study, HA secretion was decreased in the HaCaT keratinocyte culture medium after cortisol stimulation; however, it increased in response to treatment with IPE in a dose-dependent manner. Moreover, we confirmed that IPE rescued the cortisol-induced reduced expression of *HAS2* and *HAS3* in HaCaT keratinocytes (Figure 3). These results suggest that IPE may prevent cortisol-induced loss of skin moisture under psychological stress conditions. Moreover, cortisol mediates its action through the GR; after binding to the GR, the cortisol–GR complex enters the nucleus and regulates target genes. In this study, we observed that IPE blocks GR translocation into the nucleus at a level similar to a GR inhibitor (Figure 4), suggesting that IPE acts as a GR inhibitor, with a powerful inhibitory effect on cortisol activation in human keratinocytes.

This study has some limitations. Although we revealed that IPE has an inhibitory effect on the reduction in collagen and HA levels by rescuing the suppression of related genes, such as *COL1A1*, *HAS2*, and *HAS3*, and blocking GR translocation, we did not investigate the underlying molecular mechanisms of the associated transcription factors or signaling pathway molecules. Further studies are necessary to clarify the underlying molecular mechanisms by which IPE inhibits cortisol activity. In addition, we analyzed the content of IPE using HPLC-DAD analysis. The dominant component of IPE was irigenin (Figure 5), which is consistent with the finding of a previous study [32]. IPE also contained several components whose levels were considerably lower compared to the irigenin level. Further identification and bioactivity studies of these ingredients of IPE will help determine the mechanism of action of IPE against cortisol-mediated skin aging.

## 5. Conclusions

We demonstrated that IPE alleviates cortisol-induced collagen and HA degradation by regulating the expression of related genes. IPE also attenuated GR translocation, which is indispensable for cortisol action. Although further research is needed to develop IPE as a cosmetic material or herbal medicine for skin anti-aging, this study suggests IPE as an agent to prevent stress-related skin changes.

## Data Availability

The datasets used and/or analyzed during the current study are available from the corresponding author upon reasonable request.
